# Evaluation of IL-4, MIP-1α, and MMP-9 gene expression levels in peri-implant tissues in peri-implantitis

**DOI:** 10.1590/0103-6440202305382

**Published:** 2023-07-17

**Authors:** Gabriela Giro, Jorge Taira, Fernando Andriani, Sidney Watinaga, Marta Ferreira Bastos, Jamil Awad Shibli

**Affiliations:** 1 Guarulhos University, Department of Periodontology and Oral Implantology, Dental Research Division. Praça Tereza Cristina, 289, 07023-030, Guarulhos, SP, Brazil.; 2 Universidade São Judas Tadeu. Rua. Taquari, 546, 03166-000, São Paulo, SP, Brazil.

**Keywords:** Peri-implantitis, Gene Expression, IL-4, MIP-1 Alfa, MMP-9, Etiology

## Abstract

This case-control study evaluated the gene expression levels of interleukin (IL)-4, macrophage inflammatory protein type 1 alpha (MIP-1α), and metalloproteinase (MMP)-9, factors involved in the formation of giant cells in healthy peri-implant tissue and peri-implantitis. Thirty-five subjects (15 healthy and 20 with peri-implantitis), who met the inclusion and exclusion criteria, were included in this study. The peri-implant tissue biopsies were subjected to total RNA extraction, DNAse treatment, and cDNA synthesis. Subsequently, the reaction of real-time PCR was performed to evaluate the gene expression levels of IL-4, MIP-1α, and MMP-9 concerning the reference gene. IL-4 gene expression showed higher (18-fold) values in the Peri-Implantitis Group of Patients when compared with the Healthy (Control) Group (p<0.0001). Although MIP- 1α and MMP-9 gene expression levels were higher in diseased implants, they showed no significant differences (*p*=0.06 and *p*=0.2337), respectively. Within the limitations of this study, the results showed that in tissues affected by peri-implantitis, only levels of Il-4 were increased when compared with tissues in the control group.

## Resumo

Este estudo caso-controle teve como objetivo avaliar a expressão gênica dos níveis de interleucina (IL)-4, proteína inflamatória de macrófagos tipo alfa 1 (MIP-1α) e metalopreoteinase (MMP)-9, todos fatores envolvidos na formação de células gigantes em tecidos peri-implantares saudáveis e com peri-implantite. Trinta e cinco indivíduos (15 saudáveis e 20 com peri-implantite) foram incluídos nesse estudo seguindo os critérios de inclusão e exclusão. Os tecidos peri-implantares foram submetidos a extração do RNA total, tratamento de DNAse e síntese de cDNA. Subsequentemente, a reação de PCR em tempo real foi realizada para avaliar os níveis da expressão de IL-4, MIP-1α, e MMP-9 em relação ao gene de referência. O nível de expressão de IL-4 foi estatisticamwente maior (18 vezes) nos tecidos de pacientes com peri-implantite quando comparados aos pacientes saudáveis (grupo controle) (p<0,0001). Embora os níveis de expressão de MIP- 1α e MMP-9 apresentassem maiores valores nos implantes doentes, esses níveis não foram estatisticamente significantes (*p*=0.06 and *p*=0.2337) respectivamente. Dentro das limitações desse estudo, os resultados mostraram que nos tecidos afetados pela peri-implantite, apenas os nívies de IL-4 estavam aumentados quando comparados ao grupo controle.

## Introduction

Peri-implantitis is an inflammatory condition that damages both soft tissues and bone around implant-supported restorations ^1^ ^,^ ^2^. The etiology of peri-implantitis has been the subject of some debate, however, since then, it has been established that this infectious disease is associated with a complex bacterial biofilm ^3^. This pathogenic biofilm, in turn, induces the production of cytokines, increasing the inflammatory infiltrate and release of proteolytic enzymes responsible for peri-implant tissue destruction. The host’s response to producing immune-immunoinflammatory mediators in combination with dysbiosis is a key determinant in the development of peri-implant disorders ^4^ ^,^ ^5^.

However, an earlier narrative study ^6^ suggested that progressive peri-implant bone loss resulting from dental biofilm accumulation would be a misconception because this bone loss would be triggered by a foreign body reaction to titanium (Ti) particles released by the implant.

Furthermore, previous studies ^7^ ^,^ ^8^ have supported this hypothesis demonstrating that foreign body reactions could be an essential co-factor in triggering the inflammatory response to bacterial infections. Moreover, these studies have affirmed that the immune complex depends on a reaction of proteins that form part of the immune system response that recognizes foreign bodies introduced into the organism ^9^ ^,^ ^10^. Therefore, tissue injury caused by the surgical perforation of bone tissues with burs has been suggested as being a cause of infiltration of inflammatory cells that would trigger low-grade chronic inflammation. However, bone resorption attains the bone crest around an implant. The inflammation would begin to show detectable clinical signs and may eventually be compromised further, leading to implant loss ^11^ ^,^ ^12^ ^,^ ^13^.

Moreover, titanium is a chemically and biologically inert metal in contact with bone tissue, Therefore, not only has it been extensively studied since the discovery of osseointegration, but has also been the target of recent discussions about the hypothesis that the immune system would immediately recognize every Ti particle that is strange to the body. A cascade of reactions would induce modulation of the inflammation to repair the damage suffered by the body ^14^ ^,^ ^15^. Ti particles may cause damage to the mechanism of cells leading to high level of levels of reactive oxygen species expression (ROS), and increasing the levels of metalloproteinase (MMP), specifically MMP-2 and MMP9, and neutrophils recruitment. Interleukin (IL)-4, IL-6, and IL-8 levels rise as a result of Ti particle release, limiting osteoblast function, upsetting the balance of the bone, and ultimately causing bone resorption ^13^ ^,^ ^16^.

After blood-material interactions, platelets and the clot release chemoattractant are capable of attracting macrophages to the wound site, including transforming growth factor (TGF- α), platelet-derived growth factor (PDGF), CXCL4 (Platelet Factor, PF4), leukotriene (LTB4), and interleukin (IL-1). The recruitment and adhesion of macrophages at the Ti particle interface are mediated by macrophage inflammatory protein type 1 alpha (MIP-1α) ^14^ ^,^ ^16^.

In addition, Ti particles are taken up by neutrophils and macrophages, and this causes the recruitment of inflammatory cells to the surrounding tissues. Macrophage polarization determines the extent of inflammation that is caused in the surrounding peri-implant tissues. Ti particles significantly increase the total chemotaxis and inflammatory response of traditionally activated (M1) pro-inflammatory macrophages, whereas alternatively activated (M2) pro-regenerative repair macrophages are blocked ^7^.

Thus, osseointegration must also be understood as an immune-mediated inflammatory process, in which the immune system would locally regulates the repair process either positively or negatively. Therefore, this study assessed the gene expression of IL-4, MIP-1α, e MMP-9 in peri-implant tissues with or without peri-implantitis.

## Materials and methods

### Study Population

The participants included in this case-control study were 202 subjects, with implant-supported restoration in function for longer than four years, who presented for treatment or maintenance at the Oral Implantology Clinic, University of Guarulhos (UNG), Guarulhos, SP, Brazil, in the period between February 2009 and March 2013. Sample selection was based on the following inclusion criteria, as previously described ^17^: subjects with absence of lesions in the oral cavity; good oral hygiene; good conditions of health; at least one dental implant scheduled for surgical procedures for non-disease-related reasons (Healthy Group), and subjects with an implant with the indication of surgical treatment for peri-implantitis (Peri-implantitis Group). Peri-implantitis was defined as probing depth (PD) > 5mm, bleeding on probing and/or suppuration, and peri-implant bone loss >4mm ^18^.

For the Control Group, the criteria for exclusion of subjects were established, based on anamnesis, and were described as follows: smokers, patients who were excessive alcohol consumers, those undergoing chemo- or radiotherapy, with the presence of systemic diseases, blood dyscrasias, kidney diseases, diabetes, any autoimmune condition, immunosuppressed individuals, chronic users of corticosteroids or other drugs that could influence bone metabolism, pregnant or nursing mothers, or those who made regular use of oral mouthwashes, and participants who presented with peri-implantitis and/or periodontitis without treatment.

The Research Ethics Committee approved the experimental protocol of the University of Guarulhos (IRB #0007.0.132.000-10), and the volunteers signed a term of informed consent.

### Clinical Examination and Sample Collection

Clinical parameters ^2^, such as Bleeding on Probing (BoP), Suppuration, measures of Probing Depth in mm (PD), and Clinical Attachment Level in mm (CAL), were determined at six sites per implant for all the subjects included in the study. The PD and CAL measurements were recorded to the nearest mm using a North Carolina periodontal probe (PCPNU-15, Hu-Friedy, Chicago, IL, USA).

According to the approach previously outlined ^19^, two examiners completed the calibration exercise before starting with the investigation. For PD and CAL, the interexaminer variability was 0.4 mm. For the first examiner, the intraexaminer mean SE variability was 0.20 mm for PD and 0.20 mm for CAL. For the second examiner, the mean SE variability was 0.10 mm and 0.12 mm for PD and CAL, respectively. Plaque accumulation, gingival bleeding, BoP, and suppuration were calculated in the same way with two different evaluations by the k-light test (p 0.05), which takes into account the chance component of the agreement. The interexaminer agreement ranged between 0.80 and 0.95, while the intraexaminer agreement was between 0.70 and 0.95 for the first examiner and 0.80 and 0.97 for the second examiner.

### Experimental Design

Peri-implant soft tissue biopsies were taken from subjects undergoing surgical treatment for peri-implantitis (Peri-implantitis Group); and from subjects with non-disease-related conditions biopsies were performed during esthetic peri-implant surgery (Healthy group). A peri-implant soft tissue biopsy measuring 2-3 mm high and approximately 1.5 mm thick was obtained as previously described ^17^. Peri-implant tissue biopsies were obtained around dental implants from mesial or distal aspects of the peri-implant lesion (peri-implantitis group) and from the peri-implant sulci (Healthy group).

In the test group, if a single patient presented more than one diseased implant, only the worst diagnosis was included in the analysis. In the event that 2 or more implants showed a similar diagnosis, only that in the most anterior position was included in the study.

### Evaluation of Gene Expression

###  RNA Extraction 

Immediately after collection, the peri-implant tissues were incubated with RNAlater® (Ambion Inc., Austin, TX, USA) at 4^o^C for 24 hours, then samples were stored at -80^o^C until the RNA extraction process was performed. RNA samples were extracted using Trizol (Gibco BRL, Life Technologies, Rockville, MD, USA). After purification, the mRNA reverse transcription was performed (First-Strand cDNA Synthesis Kit, Roche Diagnostic Co., Indianapolis, IN, USA).

For gene expression analysis using real-time PCR, primers for GAPDH (glycerin-aldehyde-3-phosphate-dehydrogenase (reference gene) and for interleukin-4 (IL-4), macrophage inflammatory protein-1 alpha (MIP-1α) were used; primers for metalloproteinase 9 (MMP-9) levels were designed with the aid of a program developed specifically for the elaboration of *primers* for the LightCycler (Roche Diagnostics GmbH, Mannheim, Germany). All the primers were verified relative to their specificity by analyzing the Melting curve at all times using a positive and negative control. In Table 1, the sequence of primers, the profile of reactions, and amplicons generated during the responses may be visualized. Table 1 lists primer sequences, the optimized reaction profile for each primer, and the size of the amplicons.

**Table 1:** Sequence of primers, amplification profile, and estimated amplicon size.

Genes	Sequence (5’- 3’)	Amplification profile temp (°C) /time (s)	Amplicon size (pb)
IL-4	F: 5’ CCTCACATTGTCACTGCAAATC 3’	95/10, 57/7, 72/7	168
	R 5’ CTTCTGCTCTGTGAGGC 3’		
MMP-	F: 5’ GCTACCACCTCGAACTTTGAC 3’	95/10, 57/7, 72/7	248
	R 5’ CTCAGTGAAGCGGTACATAGG 3’		
MIP-α	F: 5’ GGAGTGGGTCCAGAAATATGTC 3’	95/10, 57/7, 72/7	175
	R: 5’ CTGTTTGGCAACAACCAGTC 3’		
GAPDH	F: 5’ CTGAGTACGTCGTGGAGTC3’	95/10, 56/5, 72/7	187
	R: 5’ TGATGATCTTGAGGCTGTTGTC 3’		

IL-4 = interleukin 4; MMP-9 = metalloproteinase 9; MIP-1α = Macrophage inflammatory protein-1 alpha; GADPH = glycerin-aldehyde-3-phosphate-dehydrogenase.

The RT-PCR reactions were performed using the LightCycler system (Roche Diagnostics GmbH, Mannheim, Germany) and the FastStart DNA Master SYBR Green kit (Roche Diagnostics GmbH, Mannheim, Germany). The reaction profile was determined according to the protocol suggested by the device manufacturer. DEPC water was used as a negative control for each analysis, with the reaction products quantified by using the manufacturer’s software (LightCycler Relative Quantification Software - Roche Diagnostics GmbH). The levels of GAPDH gene expression were used as a reference (housekeeping) for normalizing the values.

### Statistical Analysis

Statistical analysis was performed using the Prism 6.0 program (GraphPad Software Inc., San Diego, CA, USA). Initially, the normality of the data was analyzed using the Kolmogorov-Smirnov test. The absence of normality was found in the data regarding gene expression analysis and periodontal parameters. These data were analyzed using a non-parametric statistical method (Mann-Whitney). The results were expressed as median with minimum and maximum values. The level of significance was established at 5% (p<0.05) for all the analyses performed.

## Results

The study sample consisted of 15 participants in the Control Group and 20 in the Peri-Implantitis Group. All the individuals included in the study were shown to have healthy clinical periodontal conditions in the remaining teeth present in the oral cavity, in conformity with the inclusion and exclusion criteria of the study (data not shown).

The demographic distribution of the sample of the two Groups is detailed in Table 2. In the comparison between the two groups, no statistically significant differences in the age and distribution of genders were detected between the study groups (p>0.05).

**Table 2:** Clinical parameters (mean ± SD) and demographic characteristics of the evaluated dental implants for both groups (*n=*35). Mann-Whitney *U* and Chi-square test (*p<0.05); ns: not significant. PD: Pocket Depth, CAL: Clinical Attachment Level, BoP: Bleeding on Probing,

	Health	Peri-implantitis	***p* value**
Age (years)	49.4+5.2	48.6+5.5	ns
Gender M:F	8:7	11:9	ns
Time of loading (years)	5.2+1.3	8.3+2.4	ns
Maxilla:Mandible	6:9	8:12	ns
PD (mm)	3.42+1.32	6.88+0.11	0.001*
CAL (mm)	1.23 ± 0.54	5.58 ± 1.99	0.023*
BoP (%)	36.3 ± 13.3	80.8 ± 20.4	0.005*
Suppuration (%)	0 ± 0	19.3 ± 0.7	0.001*

During the RNA extraction process, a sample belonging to the Peri-Implantitis Group was excluded because it did not show expression of the reference gene (GAPDH). Therefore, RT-PCR analyses of the target genes totaled 15 samples for the Control and 19 for the Peri-implantitis Group.

The results showed a statistically significant difference between the Groups for expression of gene IL-4 in the comparison between the different groups of the study; with higher levels of expression (18-fold) of this cytokine in the Peri-Implantitis Group of Patients when compared with the Healthy (Control) Group (p<0.0001), as illustrated in Table 3.

**Table 3:** Levels (median+SD) of gene expression of interleukin-4 (IL-4), of the inflammatory protein of Macrophages-1 alpha (MIP-1α), and of Metalloproteinase-9 (MMP-9) in comparison with the expression of the reference gene GAPDH: glycerin-aldehyde-3-phosphate-dehydrogenase) in the peri-implant tissues. The Mann-Whitney non-parametric test (*p<0.05); ns: not significant.

Genes expression: GAPDH	Health	Peri-implantitis	***p* value**
IL-4*	0.0005+0.0020	0.0090+0.0200	0.0001
MIP-1α	0.0170+0.0230	0.0510+0.0059	ns
MMP-9	0.0022+0.0040	0.02600+0.0457	ns

In contrast, a comparison of the levels of gene expression of MIP- 1α and MMP-9 in the diseased and healthy peri-implant tissues, although higher in diseased implants, showed no significant differences (*p*=0.06 and *p*=0.2337, for MIP- 1α and MMP-9, respectively).

## Discussion

The present study demonstrated higher levels of IL-4 in tissues with peri-implantitis compared with healthy sites. These results corroborated previous findings ^20^ that revealed the presence of a higher concentration of IL-4 in the peri-implant fluid of deep sites compared with shallow areas with no bone attachment loss. IL-4 is a representative cytokine of the Th2 response family, responsible for the resolution of inflammation. Macrophages are cells that play an essential role in chronic inflammation and tissue repair. The presence of IL-4 in tissues promotes the activation of macrophages of the alternative (M2) pathway and inhibits the activation of the classical (M1) pathway; that is, the increase in repair macrophages (M2) is linked to the secretion of IL-10 and TGF-β, which result in the resolution of pathological inflammation. According to Dennison and Van Dyke ^21^, increasing IL-4 production in periodontal lesions would inhibit the activation of macrophages and proinflammatory cytokine production.

Few studies ^17^ ^,^ ^22^ have studied the characteristics of the immune response to peri-implant lesions. The lack of data demonstrating the type of reaction triggered in response to peri-implant infection has led to some authors raising different hypotheses about the pathogenesis of peri-implant bone loss, which, until recently, was believed to be triggered by biofilm in the peri-implant region.

Given the scarcity of information about peri-implant infections and the supposition that a foreign body reaction to titanium would cause peri-implantitis, this study aimed to evaluate the levels of IL-4, MIP-1α, and MMP-9 expression in an endeavor to investigate the occurrence of a foreign body reaction in peri-implant tissues affected by peri-implantitis and compare these levels with those in tissues free of peri-implant infections.

MMP-9 has been described as an essential modulator during the development of a foreign body reaction. MacLauchlan et al. ^22^ showed that MMP-9 played a pivotal role during bone remodeling and angiogenesis and regulated the process of macrophage fusion. This process showed that MMP-9 played an essential and fundamental role in functionalizing giant cells, a characteristic of the foreign body reaction. However, the results of the present study demonstrated no statistically significant difference in gene expression of MMP-9 between sites with or without peri-implantitis, contradicting earlier studies ^6^ ^,^ ^14^, which considered that marginal bone loss around implants was an imbalance resulting from a foreign body reaction to the titanium device. In addition, these studies ^6^ ^,^ ^14^ affirmed that there were two types of host responses to the insertion of a dental implant: development of a foreign body reaction, characterized by a chronic foreign body response, in which the implant is isolated from the rest of the organism by the formation of an extremely condensed bone layer; or a foreign body response to the implant would result in its fibrous encapsulation, therefore representing a primary clinical failure.

Furthermore, in this study, the authors ignored the microbial component as a possible etiological factor of peri-implant diseases, taking the survey of Donath *et al.* ^11^ as a basis. However, in the cited study, in which this author reported the foreign body reaction to implants, he did not refer to implants made of titanium. Clearly, in their study, the authors demonstrated the predominance of the presence of giant cells in the tissues analyzed, differently from the data presented in the results of the present study, in which there was no evidence of the presence of giant cells in the peri-implant tissue biopsies, therefore not characterizing the foreign body reaction. Furthermore, the studies of Fonseca *et al*. ^20^ also corroborated the results of the present study because no difference was observed in the concentrations of IL-8, a potent chemotactic cytokine produced by macrophages. It also plays an essential role during the foreign body reaction ^23^ in the peri-implant fluid sites with and without bone loss.

In addition, the results of the present study also presented no statistically significant differences in gene expression of MIP-1α in the peri-implant tissues with or without peri-implantitis. These results contradicted the finding of Petkovic et al. ^24^, who evaluated the peri-implant fluid of healthy sites, and those with initial and advanced mucositis. They observed that the beneficial sites had lower concentrations of MIP-1α than those affected by mucositis.

However, we must point out that the results from this case-control study had some limitations. First, this cross-sectional evaluation did not assess the progression of peri-implantitis, thus it did not allow a precise characterization and role of the markers evaluated in the pathogenesis and advancement of the peri-implant lesions. Secondly, a more detailed analysis of proteomics and metabolomics factors could improve the information about the entire set of proteins and metabolites. Finally, from the clinical point of view, clinicians must be aware of the well-known predisposing factors to peri-implant diseases, such as soft tissue phenotype, dental implant position, restoration design, and performing treatment planning to avoid further biological complications ^25^.

## Conclusions

Within the limitations of this study, it was possible to conclude that the gene expression of IL-4 was increased in peri-implant tissues retrieved from sites affected by peri-implantitis when compared with healthy tissues. However, no difference was observed when the levels of MIP-1α and MMP-9 expression were compared.
